# Paraform in the Treatment of Sensitive Dentine

**Published:** 1909-12-15

**Authors:** J. Ainsworth Woods

**Affiliations:** England


### PARAFORM IN THE TREATMENT OF SENSITIVE DENTINE.

BY J. AINSWORTH WOODS, L.D.S., ENGLAND.

I need make no apology, I think, for introducing the subject of the treatment of sensitive dentine to your notice. We are conscious every hour of the day of the amount of pain which may be transmitted by this tissue. I need not trouble you with any physiological speculations which have been made from time to time as to the exact method of transmitting the sensation known as pain, from, and through, dentine to the sensorium; nor need I go into the histology of the tissue, sufficient for me to know that as dental practitioners we are all perfectly familiar with the frequency with which dentine by its acute sensibility hampers our treatment, causes in many cases acute suffering during the process, and acts more than any other cause to keep the timid and nervous from having their teeth attended to and preserved.

Very many methods and preparations have been introduced with more or less success, but so far nothing, I think, promises so well as Paraform, if carefully used in suitable cases. Let me remind you, however, that it is *extremely powerful*, and must be used with the utmost caution, and with a definite knowledge of its action.

My own experience has been very limited, but so far as

I have gone I am encouraged by the results to make a more extended trial, and it will be very helpful to hear opinions formed by others who have used it.

Mr. Spiller, in a Communication read in February, 1908, before the Odontological Section of the Royal Society of Medicine, advocated its use as an obtundent; it was first introduced to his notice by Dr. Mahie, of Paris.

I will not go into the Chemistry of Paraform except to remind you that it is a polymer of formaldehyde, and that it is a white amorphous powder with a pungent odour.

Spiller advises the use of Paraform 5 parts, mixed with oxysulphate of zinc, 100 parts. This is then mixed with an aqueous solution of gum arabic to the consistency of a stiff paste. It may be covered with a temporary G. P. He says the dressing should be left in from one to seven days or longer. At the end of this time the cavity may be cut with practically no pain in nearly every case. Pain occurs from the dressing in a small percentage of cases, and is usually not localised in the tooth under treatment, it is seldom severe, and does not last long. The insensibility does not xtend to the pulp, the penetration usually being about the thickness of a thick postcard. It should not be placed in an aching tooth or too near the pulp, and if the cavity is a deep one the deeper portions should be protected from the action of the drug, the influence of which would thus be confined to the edges of the cavity.

In the discussion which followed the reading of his paper several practitioners testified to the success which had followed their use of the drug as an obtundent. Baldwin had found it not nearly so irritating as formalin.

It was also pointed out that its effects were local, and only on the dentinal fibrils exposed its influence for if it was used, say, in a cervical cavity in a molar, the dentine under a fissure cavity in the same tooth would be quite unaffected.

Cass Grayston has used a dressing composed of formaline, 1 drop; oil of cloves, 1 drop; thymol equal in bulk to one of above drops, and zinc oxide to make a paste. Sometimes this has given him very good results, but on the whole it is not very satisfactory. More recently he has been using paraffin (a quantity about the size of a pin's head), oil of cloves sufficient to dissolve it, mixed into a paste with zinc oxide and sealed into the cavity with G.P. or other temporary filling.

His experience with this is limited, and he thinks that a small proportion of paraform with a prolonged application is more satisfactory than a large dose for a shorter time.

G. G. Campion uses a form of G. P. paraform composed of

| | | |
|---|---|---|
| Paraform - - - - - | 1 part | By weight |
| Gutta percha (base plate) - - | 5 parts | |
| Plaster of Paris - - - | 7 parts | |

which he leaves in the cavity for about two weeks.

H. I. Morris has used a preparation composed of paraform 1 part, zinc oxide 10 parts, mixed into a paste with a liquid composed of equal parts of thymol and carbolic acid. He leaves this in for from four to seven days and has not found pulps irritated or any deaths of pulps resulting from the application.

My short experience—for I have only been using the drug quite recently—has been with Mr. Spiller's method in most of my cases, but more recently I have also tried Mr. Campion's G. P. paraform. I append a report of 15 cases treated.

From these results I feel distinctly encouraged to continue the use of paraform and quite agree with others that a small proportion (5% or even less) for a longer time is the best mode of application. I have certainly found that some kept ready mixed with G. P. is a very convenient form.

There is, however, the difficulty of being certain that the paraform is evenly distributed throughout the G. P. and one also wonders if in time it might not be gradually liberated, and therefore the mixture becomes less and less active.

My object, however, in writing this short communication is to gather from others their experiences and therefore improve my own methods; also to advocate an extended trial of the drug amongst those who have so far not used it, with the view that, at a later date, the combined knowledge thus obtained may be used for the benefit of those to whom we minister.—*Dental Record.*

